# Single Cell Analysis of a Bacterial Sender-Receiver System

**DOI:** 10.1371/journal.pone.0145829

**Published:** 2016-01-25

**Authors:** Tiago Ramalho, Andrea Meyer, Andrea Mückl, Korbinian Kapsner, Ulrich Gerland, Friedrich C. Simmel

**Affiliations:** 1 Physics Department, TU München, Garching, Germany; 2 Physics Department and ZNN/WSI, TU München, Garching, Germany; 3 Nanosystems Initiative Munich, Munich, Germany; Ben-Gurion University of the Negev, ISRAEL

## Abstract

Monitoring gene expression dynamics on the single cell level provides important information on cellular heterogeneity and stochasticity, and potentially allows for more accurate quantitation of gene expression processes. We here study bacterial senders and receivers genetically engineered with components of the quorum sensing system derived from *Aliivibrio fischeri* on the single cell level using microfluidics-based bacterial chemostats and fluorescence video microscopy. We track large numbers of bacteria over extended periods of time, which allows us to determine bacterial lineages and filter out subpopulations within a heterogeneous population. We quantitatively determine the dynamic gene expression response of receiver bacteria to varying amounts of the quorum sensing inducer N-3-oxo-C6-homoserine lactone (AHL). From this we construct AHL response curves and characterize gene expression dynamics of whole bacterial populations by investigating the statistical distribution of gene expression activity over time. The bacteria are found to display heterogeneous induction behavior within the population. We therefore also characterize gene expression in a homogeneous bacterial subpopulation by focusing on single cell trajectories derived only from bacteria with similar induction behavior. The response at the single cell level is found to be more cooperative than that obtained for the heterogeneous total population. For the analysis of systems containing both AHL senders and receiver cells, we utilize the receiver cells as ‘bacterial sensors’ for AHL. Based on a simple gene expression model and the response curves obtained in receiver-only experiments, the effective AHL concentration established by the senders and their ‘sending power’ is determined.

## Introduction

Components of bacterial communication systems [1, 2] have been frequently utilized for applications in synthetic biology. In an early seminal work, Weiss and Knight [3] created artificial bacterial ‘sender’ and ‘receiver’ cells based on a quorum sensing (QS) system from the marine bacterium *Aliivibrio fischeri*, which is also utilized in this work. In this system, sender cells are equipped with the *luxI* gene from the *lux* operon coding for the autoinducer synthase LuxI. LuxI catalyzes the synthesis of the quorum sensing signal N-3-oxo-C6-homoserine lactone (an acyl homoserine lactone, abbreviated AHL). AHL can diffuse through bacterial cell membranes and bind to LuxR activator proteins, which activate gene expression in receiver cells, from genes put under the control of P_lux_ promoters. In contrast to the natural QS system, in which all senders are also receivers, AHL is not utilized as an ‘autoinducer’ and there is no positive autoregulation of AHL production. Similar sender-receiver modules were already utilized in a wide variety of synthetic biology applications, e.g., in an artificial population control system [4, 5], for bacterial pattern formation [6, 7], synchronization of bacterial oscillators [8], bacterial edge detection [9], or distributed computing systems [10, 11].

In the context of synthetic biology, an important consideration is the reproducibility and robustness of synthetically generated behaviors. This is particularly challenging, as complex biological systems unavoidably display variability on various levels of organization. Over the past two decades it has become increasingly apparent that gene expression levels and their dynamics can vary considerably from one cell to another even in homogeneous colonies of genetically identical cells [12–17]. While this phenotypic heterogeneity was found to be the exception rather than the rule in a homogeneous environment [18], it is likely important for the survival of the colony in fluctuating environments. Mechanistically, the heterogeneity can be attributed to the intrinsic stochasticity of the processes involved in gene expression [19], in protein number fluctuations [20] and the noise generated by the unequal distribution of cellular components during cell division [21, 22], or other “extrinsic” factors.

The role of noise in the context of quorum sensing was previously analyzed theoretically, where in particular the impact of population feedback [23] and diffusion of the signals [24] was investigated. Diffusive coupling of the cells was surmised to lead to an overall reduction of extrinsic gene expression noise in the cells [24]. On the experimental side, quorum sensing was investigated on the single-cell level in *V. harveyi* bacteria, which communicate via two distinct autoinducer signals [25]. Noise was characterized for several reporter strains and found be extrinsic in nature. An alternative approach was demonstrated in [26], where protein level fluctuations were analyzed using correlation functions on the microcolony level rather than based on single cell data.

In contrast to previous work, we here focus specifically on an artificial sender-receiver system as typically used in synthetic biology applications. Based on fluorescence microscopy experiments [27] with bacterial cells growing in microfluidic chemostats [28, 29], we first study gene expression dynamics of a QS-derived ‘receiver module’ implemented in *E.coli*. We show that single cell data can be used to determine the quantitative input-output characteristics for the AHL/*P*_*Lux*_ system, which agree with data generated using bulk methods. We then analyze individual single cell gene expression time courses, which display a considerable heterogeneity compared to the bulk data. From these we extract statistical distributions of gene expression rates in the bacteria, and identify sub-populations with different induction beahvior. In our analysis, we first follow the time-course of the gene expression rate distribution of the whole population. By tracking individual cell lineages, we then restrict the analysis to the sub-population of the bacteria with the dominant induction state, which results in a more accurate estimate of gene expression rates and the corresponding quantitative single cell input-output characteristics.

Finally, we apply our analysis procedure to a synthetic sender-receiver system [3, 11], in which the AHL signals are produced *in situ* by dedicated sender bacteria. In order to be able to determine AHL concentration within the chambers, we utilize the highly sensitive receiver cells themselves as AHL bioreporters [30]. Using a simple model of gene expression in sender and receiver bacteria, we can deduce the effective AHL concentration established by the bacteria in the microchambers, which falls in the low nanomolar range for our experimental setup. We show that the effective AHL concentration scales with the average sender/receiver ratio in the chamber and rises ∼*t*^2^ with time.

A similar analysis could help to provide a quantitative experimental basis for the ongoing debate about the evolutionary origins of QS [31, 32]. The question is centered around whether the autoinducer is really a social signal to other cells, or instead simply a single cell mechanism to measure effective diffusion in the local environment. This distinction is relevant in the context of heterogeneous colonies, where different individuals may evolve to respond differently to the autoinducer—if some contribute external molecules which benefit all cells while others do not, then ‘cheaters’ can have a growth advantage. The analysis approach established here for the synthetic sender-receiver system will be useful to quantitatively characterize the QS behavior by controlling effective AHL diffusion as well as population sizes.

## Materials and Methods

### Plasmids

All experiments were performed with genetic constructs derived from the *Aliivibrio fischeri* bacterial quorum sensing system (cf. Fig A in S1 File). Receiver plasmids contained the *luxR* gene under control of the TetR repressed promoter P_tet_ and the *gfpmut3b* gene [33] controlled the lux promoter P_lux_ (BioBrick part BBa_T9002) on vector pSB1A3. In the absence of TetR, LuxR was constitutively expressed from this plasmid. To construct the sender plasmids, the gene for LuxI synthase (BioBrick part BBa_C0261) was cloned into a pETDuet-1 expression vector (Merck Millipore) inserted between the BioBrick cloning sites XbaI and PstI. Expression from this vector is driven by T7RNAP, which is produced by the compatible host strain E. coli BL21(DE3)pLysS after IPTG induction. As a fluorescent reporter, an additonal *rfp* gene (derived from BioBrick BBa_E1010) was cloned between the NdeI and PacI restriction sites of the plasmid. A complete description of the construction of the plasmids including their sequences can be found in a previous publication [11]. Receiver and sender cells were created by transforming the corresponding plasmids into *E. coli* BL21(DE3)pLysS using an Electroporator (ECM399, BTX Harvard Apparatus, Holliston, MA, USA).

### Bacterial cell culture

Experiments were performed with the *Escherichia coli* strain BL21(DE3)pLysS (Promega, Fitchburg, WI, USA). Cells were grown in 10 ml Luria-Bertani (LB) medium (Carl Roth, Karlsruhe, Germany), containing 100 *μ*g/ml Carbenicillin (AppliChem GmbH, Darmstadt, Germany) and stored for 4 hours in a shaker (Innova 44R, New Brunswick scientific, Edison, NJ, USA) at 37°C and 250rpm. After 4 hours, the OD600 typically was between 1.0 and 1.5. The OD600 was then adjusted to 1.0 with fresh LB medium. 10 ml of the culture were centrifuged for 5 minutes at 7000 rcf. The supernatant was decanted and the remaining pellet was resuspended with 1 ml LB medium for the microscopy experiment and 10 ml LB medium for plate reader measurements.

### Plate reader experiments

Bulk characterization of gene expression activity was performed in a FLUOstar Omega plate reader (BMG, Ortenberg, Germany). A 96-well plate (ibidi, Martinsried, Germany) was prepared by combining 30*μ*l of a 10 X AHL stock solution (corresponding to the desired 1 X concentration) and 240*μ*l LB medium. 30*μ*l of bacterial suspension in LB were added directly before the experiment was started. Fluorescence and optical density were measured every 5 minutes for 15 hours. Between two consecutive measurements the plate was shaken with 1100 rpm. To prevent evaporation a gas permeable laminate (Carl-Roth GmbH, Karlsruhe, Germany) was bonded onto the plate. Fluorescence was excited at *λ*_*exc*_ = 485 nm for excitation and detected at *λ*_*em*_ = 520 nm. The absorbance of the cell suspension was measured at 600 nm. Cells were initially diluted to an absorbance of OD600 = 0.1, and inducer was added at concentrations ranging from 0 nM to 100 nM. AHL did not have any observable influence on bacterial growth during exponential phase, while it slightly affected the saturation level. Bacterial cultures appeared to grow to higher densities at higher inducer concentration.

### Microfluidic chemostats

Microfluidic chemostats consisted of bacterial traps (100 *μ*m × 60 *μ*m × 1 *μ*m) connected to microfluidic supply channels (*width* × *height* = 100 *μ*m × 15 *μ*m), similar to those previously described in Ref. [8]. AHL concentrations were varied using a microfluidic gradient mixer adopted from [34]. A schematic design of the microfluidic device is shown in Fig B in S1 File. The chemostats were fabricated using standard soft lithography procedures. A lithographic master was first defined by photolithography on a silicon wafer using the negative resists EpoCore 20XP (micro resist technology, Berlin) and AZ-nLOF 2070 (Microchemicals, Ulm, Germany). Channels and traps were defined separately in two consecutive steps. The microfluidic channels were then molded in the elastomer Polydimethylsiloxane (PDMS) (Sylgard 182, Dow Corning, Seneffe, Belgium). After baking for 2 h, PDMS and a microscopy cover glass slide were sonicated for 10 minutes in 2/3 isopropanol and 1/3 ddH_2_O, followed by exposure to an oxygen plasma in a plasma cleaner (Femto, Diener electronic, Ebhausen, Germany) for 1 minute, after which the PDMS was bonded to the glass. Calibration measurements using fluorescent buffer solution (cf. Fig C in S1 File) showed correct performance of the gradient mixer with a relative precision in the concentrations of ±20%.

For the experiments, bacterial suspension was flushed through the microfluidic system until single or few bacteria were captured in the traps. After trapping, bacteria were constantly supplied with nutrients (LB medium) using a syringe pump with two syringes at a speed of 2× 80*μ*l/h. For the titration experiments, AHL (N-3-oxo-C6-homoserine lactone, Sigma Aldrich, Taufkirchen, Germany) was added to the medium to achieve the desired concentrations after passing the microfluidic mixer. The bacterial growth rates in the microfluidic chambers ranged from *μ* ≈ 0.36 h^-1^ up to *μ* ≈ 1 h^-1^, independently of the inducer concentration.

### Fluorescence Microscopy

Time-lapse microscopy was performed on an automated fluorescence microscope (IX81, Olympus, Tokyo, Japan) equipped with a ZDC2 laser autofocus system and a motorized x-y-stage (Scan IM, Maerzhaeuser, Wetzlar, Germany). The microscope was enclosed in a cage incubator (okolab, Ottaviano, Italy) and held at a constant temperature of 37°C. Brightfield and fluorescence images were acquired every 3 minutes for 15 hours with an emCCD camera (iXon3 888, Andor, Belfast, UK) through an oil-immersion 100x objective (UPlanSApo 100x, Olympus, Tokyo, Japan) with acquisition times of 0.2 seconds. and all devices were controlled via CellSense software (Olympus, Tokyo, Japan). Fluorescence was excited by an x-cite 120 lamp (EXFO, Quebec, Canada).

### Image analysis and extraction of single cell data

A detailed description of the image processing procedures is found in S1 File. In brief, microscopic images are first preprocessed by applying contrast enhancement, noise reduction, and sharpening algorithms (Fig D in S1 File). As a second step, each pixel is classified as belonging to a cell or not, based on a hybrid method: global brightness, adaptive local brightness and an adaptive masking method (adapted from Wang et al. [35]) each contribute to the classification of a pixel as ‘cell area’ or ‘image background’. After removal of the background pixels, cell markers are created by a multi-step process. First, connected regions of cell area pixels are assigned to a shared marker. These regions are then broken down based on edge information, brightness, and cell geometry until all regions have dimensions consistent with the cell type. The resulting regions are refined using the watershed algorithm. Spurious results may be removed by filtering regions by size and via the use of a classifier. We use a support vector machine (SVM) as classifier, which has previously been used to distinguish cell phenotypes with success [36]. The classifier is trained by the user via a graphical user interface (GUI). Finally, cells are tracked in time using a maximum-overlap method. This method compares cell labels in two adjacent frames and calculates their overlap. For each cell in the later frame, the cell with maximum overlap in the previous frame is assigned as its parent, and cell lineages can be constructed from the data (Fig E in S1 File). The user can correct tracking assignments via the GUI.

### Data analysis

In plate reader experiments, the time-dependent optical density (OD600) is taken as a proxy for the total cell mass, *M*(*t*). Together with the fluorescence *F*(*t*) the expression rate *α* is calculated via

$$\begin{array}{c} \alpha ∼\dot{F}/M\;. \end{array}$$ (1)

This proportionality holds for expression of a stable fluorescent protein during exponential growth of the bacteria: In exponential growth, the total mass grows according to $\dot{M}=\mu M$, while the number of fluorescent proteins *p* per bacterium follows $\dot{p}=\alpha -\mu p$, which assumes that protein concentration is only diluted by bacterial growth. Since *p* ∼ *F*/*M*, one has

$$\begin{array}{c} \frac{d}{dt}\frac{F}{M}=\frac{\dot{F}}{M}-\frac{\dot{M}F}{M^{2}}=\frac{\dot{F}}{M}-\mu \frac{F}{M}, \end{array}$$ (2)

and thus

$$\begin{array}{c} \frac{\dot{F}}{M}=\frac{d}{dt}\frac{F}{M}+\mu \frac{F}{M}\;, \end{array}$$ (3)

which is proportional to $\dot{p}+\mu p=\alpha$. In plate reader data analysis, we took the maximum of $\dot{F}/M$ during exponential growth as an approximate measure for *α*. In microfluidic experiments, the area *A* occupied by the cells takes the role of the cell mass/absorbance in the bulk experiments and thus $\alpha ∼\dot{F}/A$.

## Results and Discussion

### Gene induction by AHL—population average

We first characterized the average gene expression response of our receiver cells using standard plate reader experiments. Receiver cells constitutively expressed LuxR and thus, in the presence of the QS inducer AHL, produced the fluorescent reporter protein GFPmut3 (Fig A in S1 File). Experiments with varying inducer levels ([AHL] = 0–100 nM) were used to deduce the response curve of the bacteria, which was well fit by a Hill function with Hill exponent *n* = 0.97 ± 0.08. The AHL concentration required for half induction was obtained from the fit as *K* = 13.9±1.7 nM (cf. Fig F in S1 File). These parameters are consistent with previous quantitative analyses of the AHL/P_lux_ system, which have typically resulted in Hill exponents around *n* = 1–1.5 and induction thresholds in the range *K* = 5–15 nM [25, 37–39].

Using time-lapse fluorescence microscopy [27], we then recorded the response of growing populations of receiver bacteria within microfluidic bacterial chemostats similar to those previously described in [8] (see Methods and Fig B in S1 File). In these structures, bacterial cells are captured in shallow microchambers of dimensions 100 *μ*m × 60 *μ*m and ≈ 1*μ*m height, which only allow cell growth in a single layer. The microchambers are connected to larger microfluidic supply channels, which continuously provide fresh medium and remove waste products from the chambers. As a result, bacteria can grow in the chambers in exponential phase over extended periods of time.

**Fig 1:**
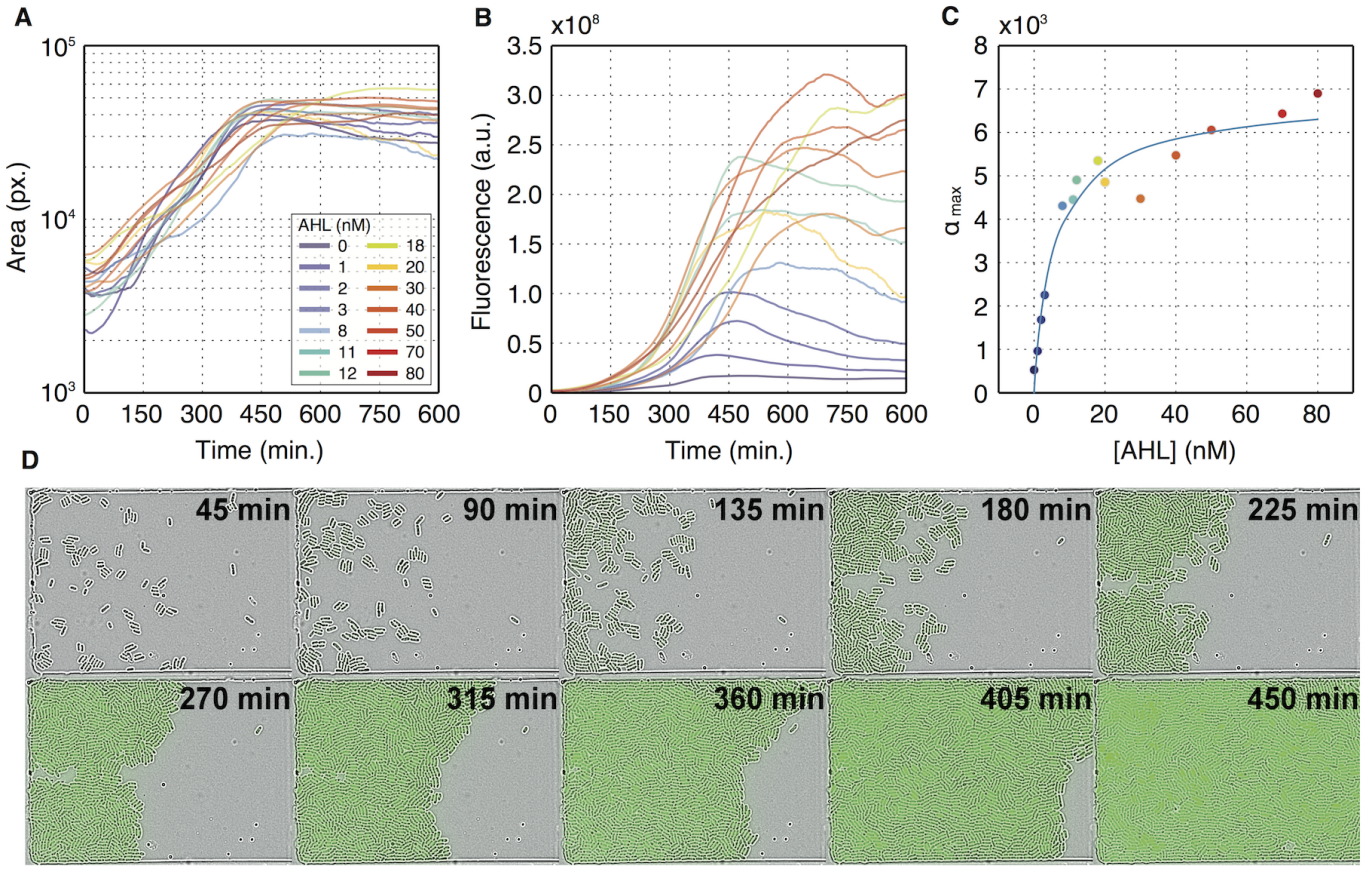
Bulk analysis of gene expression in microfluidic chemostats. (A) Total cell area *A* in pixels as a function of time for acquisitions with different AHL concentrations. The cells are in exponential growth for at least 450 *min*. After this time, the bacteria completely fill the microfluidic traps and the measured area stays constant. (B) Total colony fluorescence *F* as a function of time. (C) Maximum gene expression rates calculated as $\dot{F}/A$ (cf. Eqs 1–3). The solid response curve is a Hill fit to the data with *n* = 0.95±0.2 and *K* = 5.3±1.4 nM. (D) Snapshots taken from a time-lapse microscopy video of a bacterial colony growing in a microfluidic trap, which is connected to a supply channel on the right. The images shown are overlays of bright-field and fluorescence data. In the example, the bacteria were induced with 12 nM AHL.

In the experiments, we recorded bright field (BF) and fluorescence images of growing bacterial populations over a timespan of typically 15 h with a temporal resolution of 3 minutes. The microscopy images were then analyzed using a custom-written image analysis software package, which is described in detailed in the Supporting Information (Text A in S1 File). We first used the extracted data to determine the colony average of all observables, permitting us to compare the average response in the microfluidic chemostats to the bulk response measured with the plate reader. Fig 1A shows the time-dependent total area *A*(*t*) of all cells in a microcolony for different external AHL concentrations, while Fig 1B shows the total integrated fluorescence *F*(*t*) for a colony. Since the total area is a proxy for total cell mass, the average gene expression activity *α* for the microfluidic experiments can be defined as $\alpha =\dot{F}/A$. Taking *α*_max_ from each curve, we can construct the average response of the AHL/P_lux_ system (Fig 1C). In this case, the Hill function fit leads to a cooperativity exponent of *n* = 0.95±0.2 and half activation at *K* = 5.3±1.4 nM. The Hill exponent thus agrees well with the one extracted from the bulk experiment, while the induction threshold *K* is somewhat lower in our microfluidic device. This difference could be caused by the different physiological state of the bacteria in the microfluidic environment, e.g., their significantly lower growth rate compared to the bulk experiment. Additionally, the microfluidic setup may introduce a small deviation between the expected and actual local AHL concentration (Fig C in S1 File).

### Analysis of gene expression variability

**Fig 2:**
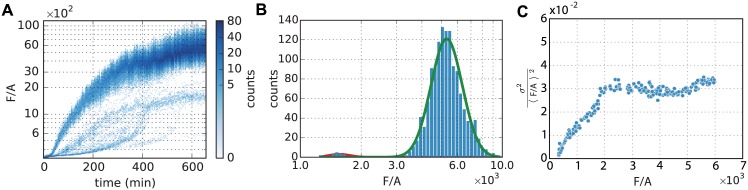
Single cell gene expression histogram and trajectories for AHL = 50 nM. (A) Evolution of the bacterial fluorescence per area *F*/*A* as a function of time. Clusters of ‘late inducers’ are visible. (B) Histogram of *F*/*A* at time *t* = 600 min using a logarithmic scale on the x-axis. Solid lines represent the two Gaussian distributions resulting from the Gaussian mixture fitting procedure used to separate out the dominant, homogeneous fraction of cells. (C) Parametric plot of the square of the coefficient of variation (CV) of *p* = *F*/*A*, i.e., $\sigma_{p}^{2}/{\langle p\rangle }^{2}$, as a function of 〈*p*〉. The noise is dynamically ramping up until the cells reach roughly 1/3 of their maximal expression level. After this, *CV*^2^ stays approximately constant, indicating the dominance of extrinsic noise in gene expression.

We next characterized the stochastic response of the AHL/P_lux_ system by extracting time-dependent histograms of fluorescence levels from our data. Fig 2A shows an example for such a time-dependent histogram where the cells were induced with 50 nM AHL. Note that in this analysis the identity of the individual cells is not followed. From a theoretical perspective, this characterization of the gene expression dynamics corresponds to the Fokker-Planck description of stochastic systems in terms of a time-dependent probability distribution, in contrast to the Langevin description in terms of stochastic trajectories (see below).

In order to display the entire range of expression levels, we plot the fluorescence per cell area in Fig 2A on a logarithmic scale. A single time slice, taken at the late time point *t* = 600 min, is displayed in Fig 2B. The Gaussian fit to the main peak (green line) shows that the dominant part of the gene expression histogram is well described by a Gaussian distribution on the logarithmic axis, which corresponds to a lognormal probability distribution for the expression level. Theoretically, a lognormal distribution is expected to be a good description for a biological quantity that is determined by several independent kinetic rates. For instance, if the steady-state concentration *p* of a protein is determined by the rates of mRNA synthesis, *α*_*r*_, and degradation, *λ*_*r*_, as well as the translation rate *α*_*p*_ and the rate of protein degradation *λ*_*p*_ via *p* = *α*_*r*_ *α*_*p*_/*λ*_*r*_ *λ*_*p*_, and the statistical variations of these rates from cell to cell are not strongly correlated, then the central limit theorem can be applied to the logarithm of this expression [40, 41]. The theorem states that the limiting distribution obtained for many different rates is the lognormal distribution, but in practice the distribution will already be very close to lognormal even when only a couple of rates are involved, as in this example where *p* is determined by four rates. Previously, a variety of distributions for gene expression variability have been theoretically derived [19, 20, 42–44] from different assumptions for the underlying noise process, including the negative binomial and the Gamma distribution, which are empirically hard to distinguish from the lognormal distribution [41].

Whereas the dominant part of the distribution in Fig 2B is well described by the lognormal distribution, there are evidently other contributions at lower expression values. The time-dependence of this contribution is visible in Fig 2A and suggests that the population contains a small group of bacteria, which respond much later and less strongly than the majority. We hypothesized that this fraction of cells is in a different physiological state, which appears consistent with the observation that the number of these cells does not grow significantly in contrast to the cells in the dominant part of the distribution, see Fig 2A. We therefore separated out these slow-growing ‘late-inducers’ from the dominant induced population using a Gaussian mixture model for the logarithm of the expression data (in Fig 2B, this is shown by the red and green lines). This procedure allowed us to extract, at each time point, the mean and variance of gene expression within the dominant part of the cell population, which appears to be homogeneous in its physiological state.

We next analyzed the noise characteristics within the dominant cell population (Text B in S1 File). From the mean, 〈*p*〉, and the variance, $\sigma_{p}^{2}$, of protein expression, we calculated the fractional noise $\sigma_{p}^{2}/{\langle p\rangle }^{2}$, which corresponds to the square of the coefficient of variation *CV* = *σ*_*p*_/〈*p*〉. This is plotted in Fig 2C against the mean expression level. Note that Fig 2C is a parametric plot, where both the fractional noise and the mean are functions of time. It indicates that $\sigma_{p}^{2}/{\langle p\rangle }^{2}$ is approximately constant after reaching about one third of the maximal expression, i.e. from this point on the standard deviation increases proportional to the mean. The obtained *CV* ≈ 0.17 is about half of that obtained previously for *V. harveyi* autoinducer reporter systems [25]. The scaling of the fractional noise with the mean is often used to distinguish between intrinsic and extrinsic noise contributions. A recent high-throughput study with *E. coli* suggested that generally intrinsic noise is dominant at low expression levels, while extrinsic noise is dominant at high expression levels [45]. Our observation of constant fractional noise at high expression levels is consistent with the scaling expected for extrinsic noise.

At expression levels below one third of the maximal expression, the fractional noise in Fig 2C is not constant but increases roughly linearly with the mean. This behavior is neither consistent with extrinsic noise nor with intrinsic noise, which would predict a fractional noise that decreases with the mean. Strictly speaking, the scaling laws for intrinsic and extrinsic expression noise only apply to the steady-state, while the increasing regime of Fig 2C corresponds to the time period during which gene expression is dynamically ramping up. Nevertheless, the increasing fractional noise appears somewhat surprising given that existing models for time-dependent noisy gene expression [44] rather display a decrease in the fractional noise as the mean expression level rises. However, the precise stochastic dynamics of the initial induction process likely depends on many details including the dynamics of the reporter system, and it is unclear whether this period leads to any generic features that can be captured by a simplified mathematical model. In contrast, the last two thirds of the induction process nicely follow the generic extrinsic scaling.

### Extraction of response curves from single cell trajectories

Up to now, we characterized the induction response of our bacteria only on the *colony level*. We first focused on the temporal evolution of the mean gene expression (Fig 1), and then determined the statistical variation of gene expression levels within the population (Fig 2). The latter analysis demonstrated that the population is in fact quite heterogeneous, and thus the response of individual cells potentially could be different from the mean behavior.

We therefore tracked the gene expression dynamics of individual cells and determine the response curve of a single homogeneous subpopulation (for Details on Video Analysis cf. Text A in S1 File) and the establishment of Single Cell lineages (Fig E in S1 File)). In order to filter out cells with a specific induction behavior, we classified cells via their expression level at the end of the experiment and excluded trajectories of all cells outside of the targeted subpopulation. From the fluorescence time traces we then calculated the full temporal dynamics of the production rate *α* for each cell. As before, we utilized the maximum of this value (max_*t*_ *α*(*t*)) as a measure for the induction level of the cells. As an alternative measure, we also calculated the mean production rate 〈*α*(*t*)〉_*t*_ for each cell.

**Fig 3:**
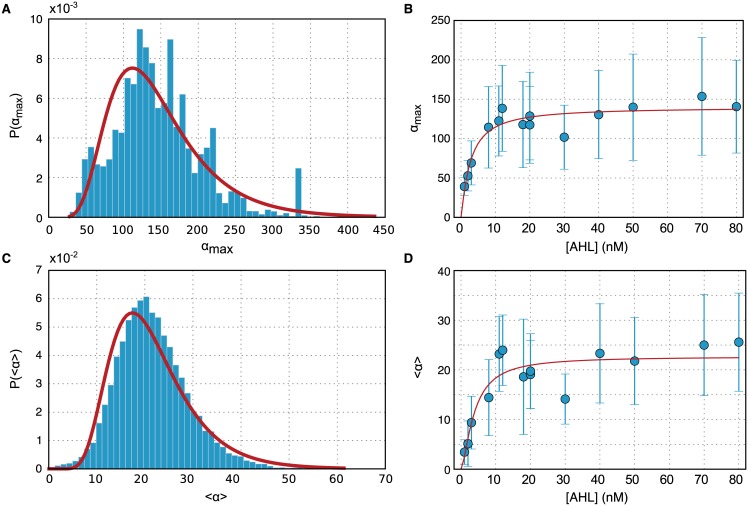
Distribution of gene expression rates *α* determined from single cell trajectories. (A) Histogram of the maximum gene expression *α*_*max*_ = max_*t*_ *α*(*t*) for each single cell trajectory (AHL = 50 nM), and a lognormal MLE fit to *P*(*α*_*max*_) (red line). (B) Plot of the average and standard deviation of the distribution *P*(*α*_*max*_). The red line is a regression curve generated by fitting a Hill function to the data. (C) Histogram of the average expression 〈*α*(*t*)〉 for each single cell trajectory (AHL = 50 nM) and a lognormal MLE fit to *P*(〈*α*(*t*)〉). (D) Plot of the average and standard deviation of the distribution *P*(〈*α*(*t*)〉)—the red line represents the best fit with a Hill curve.

In Fig 3 we compare the distributions obtained for each observable, which are both fit well by a lognormal distribution. We extracted the average and standard deviation of these distributions as a function of AHL concentration. This allowed us to plot induction response curves such as in Fig 1C, but now based on single cell data—and with error bars. For max_*t*_ *α*(*t*), a fit with a Hill curve results in *K* = 2.7±0.6 nM and *n* = 1.2±0.3, while for 〈*α*(*t*)〉_*t*_ we obtain values of *K* = 3.8±1.2 nM and *n* = 1.5±0.6.

The latter values thus represent the induction threshold and Hill exponent obtained from the average expression rates of single bacteria belonging to a single subpopulation, and should therefore be the ‘most reliable’ estimate of these parameters for our system. While the obtained values are in agreement with those fit to the bulk response curve (Fig 3C) within statistical error, we observe that the predicted response is at a higher cooperativity and lower threshold. This is consistent with the fact that the late inducer population effectively lowers the observed average fluorescence per mass unit, resulting in a (predicted) more gradual response curve in the bulk case. Focusing only on quickly induced cells we obtain a sharper response, suggesting that the heterogeneity in the population smoothes out the response curve.

### Quantification of AHL concentrations in a sender-receiver system

**Fig 4:**
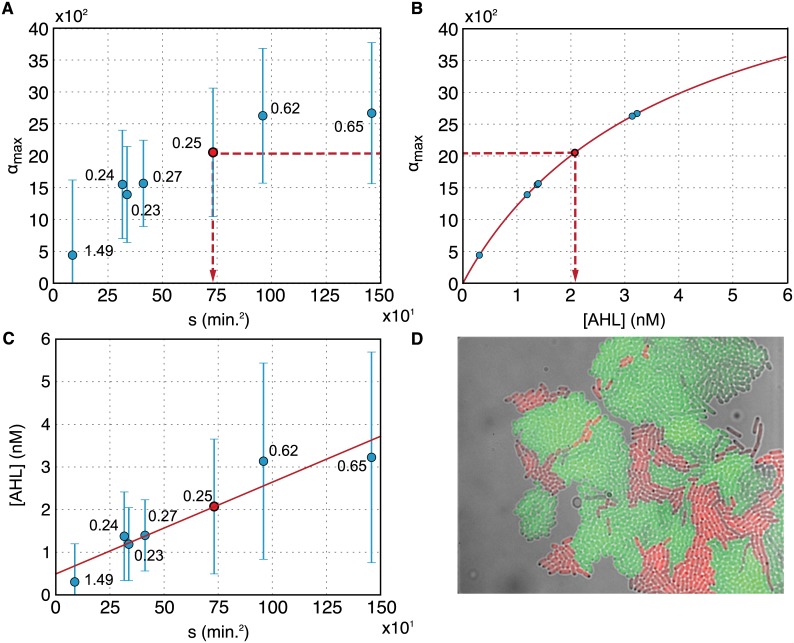
(A)Maximum GFP induction for 7 select experiments as a function of the effective AHL production constant $s=\langle r\rangle t_{\text{max}}^{2}$. The sender strength parameter *s* in the experiments was varied via the sender/receiver ratio *r*. Nominally, the initial ratios were chosen to be *r* = 0.067, 0.142, 0.33, and 1 (corresponding to sender *fractions* of 6.25%, 12.5%, 25%, and 50%, resp.), but due to cell division and dynamics within the bacterial traps, the ratios varied over time—the resulting average ratios 〈*r*〉 are indicated for the single data points. Error bars represent standard deviations obtained from single cell gene expression histograms as in Fig 3. (B) Determination of effective AHL concentration for the 7 experiments using the calibration curve acquired in the ‘receiver only’ experiments with constant [AHL]. (C) The parameter *s* and the effective AHL concentration in the traps can be related as indicated by the broken lines in part A and B of the figure (for the red colored example point). A linear fit to the data following Eq (6) is shown as a red line, error bars are obtained from the standard deviations in (A) via error propagation. (D) Example image of a microfluidic trap containing sender (red) and receiver (green) bacteria.

We next attempted to quantify the chemical communication between bacterial sender and receiver cells within the microfluidic chambers (Fig 4). While receiver cells were the same AHL sensing bacteria as described above, sender cells were equipped with a plasmid containing a gene for LuxI, an AHL-synthase, and red fluorescent protein (RFP) as a fluorescent marker. Thus the sender cells were capable of locally producing a QS signal, which can spread into the microfluidic environment and induce GFP expression in the receiver cells. We performed a series of experiments, in which we loaded small numbers of senders and receivers into chemostat microchambers at varying initial ratios *r*, and monitored their growth and communication using time-lapse microscopy as before.

A quantitative analysis of these sender-receiver experiments is complicated by a variety of issues. First, there is no simple sensor available for *in situ* sensing of AHL except for the receiver bacteria as ‘cellular sensors’ themselves. Furthermore, both the senders and receivers are growing and dynamic, and thus at any given time the signal output depends on the history of the system and the specifics of the experiment (such as sender/receiver ratio *r*, growth and expression rate). In order to determine the effective AHL concentration (or ‘sender strength’) for each experiment individually, we therefore have to resort to a model of gene expression dynamics in the system.

In the model, the production of the AHL synthase LuxI is described by

$$\begin{array}{c} \frac{d}{dt}\text{[LuxI]}\left(t\right)=\alpha_{l}\;rN\left(t\right)-\lambda \;\text{[LuxI]}\left(t\right). \end{array}$$ (4)

Here *α*_*l*_ is the LuxI production rate, *N* is the total number of receiver bacteria and [LuxI] is the mean concentration of LuxI molecules in the microfluidic chamber at time *t*. The rate *λ* accounts for degradation/dilution of LuxI, and *r* is the sender/receiver ratio, which can be different in each experiment and due to cell division may vary over time. AHL is then produced from LuxI with rate *α*_*a*_ and distributes within the chamber through diffusion. We further neglect AHL decay, but assume a constant outflow from the chamber proportional to *C*:

$$\begin{array}{c} \frac{d}{dt}\text{[AHL]}\left(t\right)=\alpha_{a}\text{[LuxI]}\left(t\right)-C\;\text{[AHL]}\left(t\right) \end{array}$$ (5)

In the exponential growth phase—when *N*(*t*) = *N*_0_ *e*^*γt*^-, the above equations can be solved for [AHL] analytically. For small enough growth, degradation, and dilution rates (*γ*, *λ*, *C*), the resulting expression for the concentration [AHL] at time t can be approximated by [AHL](*t*) ≃ 1/2*α*_*a*_ *α*_*l*_ *rN*_0_ *t*^2^ (cf. Text C in S1 File). Intuitively, this can be understood as follows: at any time *t* there will be a number of sites producing AHL proportional to *t* (due to population growth). Integration with respect to time results in a total AHL production in the chamber proportional to *t*^2^. The proportionality constant (*α*_*a*_ *α*_*l*_ *rN*_0_/2) depends on the AHL and LuxI production rates and the initial sender population size.

The sender-receiver ratio *r* and the times *t* at which AHL concentration is measured are not constant from experiment to experiment. For each experiment we therefore fix the measurement time *t* to be the time at which senders are maximally induced (*t*_max_), and the sender-receiver ratio is estimated by averaging *r*(*t*) in the interval [0, *t*_max_]. In order to be able to compare different sender-receiver experiments, we bundle these experimental parameters into the variable $s=\langle r\rangle t_{\text{max}}^{2}$. This procedure allows us to set up a calibration curve that relates the parameter *s* to the effective amount of AHL in the chamber:

$$\begin{array}{c} {\text{AHL}}_{\text{eff.}}\left(s\right)=\frac{1}{2}\alpha_{a}\alpha_{l}N_{0}s \end{array}$$ (6)

Under the assumption that AHL diffuses quickly through the microchamber (estimates for its diffusion coefficient *D* are in the range from 100 to 1000 *μm*^2^/*s* [9, 46–48]) such that each cell is exposed to the same concentration [AHL], the GFP expression rate of the receiver cells is given by:

$$\begin{array}{c} \alpha \equiv \frac{d}{dt}\text{[GFP]}\left(t\right)=\alpha_{g}\frac{{\text{[AHL]}}^{n}\left(t\right)}{{\text{[AHL]}}^{n}\left(t\right)+K_{g}^{n}}, \end{array}$$ (7)

where *n* and *K*_*g*_ are the Hill exponent and threshold for AHL induction determined above.

We can now match any experimentally determined GFP expression rate *α* to a parameter *s* characterizing each experiment (Fig 4B). At the same time, *α* can be matched to an effective AHL concentration via Eq (7) (Fig 4C). As explained in Fig 4D, this also establishes a relationship between AHL and the parameter *s*. which can be used to characterize the sender strength of the sender bacteria according to Eq (6). In practice, we might want to determine the ‘effective’ AHL concentration in a sender-receiver experiment, compare data from two different experiments, or we might ask whether two experiments are comparable at all. Our results indicate that this could be done using a similar procedure as that detailed above, i.e., via determination via a parameter *s* that depends on the sender ratio in the population and grows *quadratically* with time.

## Conclusions

We have quantitatively studied gene induction by the diffusible quorum sensing inducer N-3-oxo-C6-homoserine lactone in genetically modified receiver bacteria, in which the expression of green fluorescent protein was put under the control of the quorum sensing promoter P_lux_. In order to characterize gene expression dynamics on the single cell level, we performed experiments in microfluidic chemostats and monitored bacteria by fluorescence video microscopy. Using customized image analysis software, we were able to track large numbers of bacteria over extended periods of time, determine bacterial lineages and filter out subpopulations within a heterogeneous population.

We then quantitated the single cell response of bacteria to varying amounts of inducer using different methods. We followed the temporal evolution of the full statistical distribution of gene expression activities in bacterial populations, which allowed us to identify several subpopulations of bacteria with distinct induction behavior. Response curves derived from the mean behavior of the microchamber populations agree well with those obtained in bulk gene expression, except for a lower induction threshold which is attributed to the different growth conditions in the chemostat. We also constructed response curves from single cell trajectories, which enabled us to focus on the dominant sub-population with homogeneous induction behavior. Analysis of this homogenous, major sub-population resulted in a slightly steeper response to the autoinducer (larger Hill exponent) than for the whole population. Small numbers of cells appeared to respond much later to added AHL, which could be attributed to a strongly reduced growth rate. Gene expression noise in receiver bacteria was found to be extrinsic in nature, consistent with previous studies of other bacterial communication systems. Somewhat surprisingly, the coefficient of variation was found to dynamically ramp up in the initial (non-steady state) phase of growth in the chamber, but remained constant after approximately 1/3 of the maximum gene expression was reached.

We also applied our methodology to the characterization of bacterial microchambers containing both AHL senders (expressing the autoinducer synthase LuxI) and receivers, where we used the receivers themselves as highly sensitive bioreporters for AHL. Based on a simple gene expression model of the sender-receiver system and the response curves obtained in the receiver-only experiments, we were able to determine the effective AHL concentration established by the senders in the microchambers, and also assign an effective ‘sender strength’ to them. The sender strength can be adjusted by the sender/receiver ratio in the chambers, but due to statistical fluctuations this ratio can fluctuate over time and vary from experiment to experiment. In addition to this ratio, the effective AHL concentration in a chamber is approximately proportional to *t*^2^, which is due to the combined effect of sender cell growth and simultaneous AHL production.

Taken together, we quantified both the response of the receiver cells as well as the emitting power of sender cells on the colony and single-cell level. This contributes a better characterization of this important inter-bacterial communication channel for rationally designed synthetic biology applications that takes the stochastic nature of gene expression into account. The same approach and methods can be used to characterize natural quorum sensing systems in quantitative detail to further elucidate the communication behavior in bacterial communities.

## Supporting Information

S1 File Supplementary Text A–C, Supplementary Figs A–F. Text A, Image processing. Text B, Gene expression noise. Text C, Sender—receiver system. Fig A, Schematic overview of the bacterial sender-receiver system. *Sender cells*: As indicated, in the presence of IPTG repressor protein LacI is not bound to the lac promoters *P*_*LacUV*5_ on the bacterial genome and *P*_*T*7*lac*_ on the sender plasmid. T7 RNA polymerase is then expressed, which in turn leads to the expression of AHL synthase LuxI and fluorescent reporter protein RFP from the plasmid. LuxI catalyzes the production of the quorum sensing signal N-3-oxo-C6-homoserine lactone (AHL), which can freely pass through the bacterial cell wall. *Receiver cells* constitutively express activator LuxR from the receiver plasmid. In the presence of AHL, LuxR activates GFP expression, which is under the control of the lux promoter *P*_*lux*_. In the first set of experiments in the main paper, only receiver cells are used and AHL is manually added to the culture medium to induce gene expression. Fig B, Microfluidic chemostats. (A) The microfluidic chemostat consists of a gradient mixer (adopted from Ref. 55 of the main paper), which generates linear concentration gradients of chemicals supplied through inlets 1 and 2, respectively. Eight gradient exits are connected to a total of 2 × 8 microfluidic channels, which contain trapping regions for bacteria (similar to Ref. 8 of the main paper). In the experiments, the concentration of AHL was varied in 1 nM steps in the range 0–21 nM, and in 10 nM steps in the range 20–90 nM. (B) Top view of a supply channel (blue) with trap region (grey). (C) Side view (not drawn to scale) showing the reduced height of the trap region, which only allows bacterial growth in a single layer. Fig C, Calibration of the gradient mixer system. We performed a series of calibraton experiments (with flow rates 40, 80, 160 and 320 *μ*l/h) to evaluate the quality of the concentration gradient generated by the microfluidic mixer shown in Fig B in S1 File. In these experiments the right reservoir was loaded with buffer solution containing 10 *μ*M fluorescein and the left reservoir with pure buffer (0 *μ*M). After establishment of a steady gradient, we measured the fluorescence in the trap regions. The background-subtracted fluorescence values were then plotted against the nominal concentrations expected for the traps. As shown in the figure (which is obtained for the 160*μ*l/h case), indeed a linear concentration gradient is generated. A linear regression fit to these values (fixed at 0*μ*M) allows us to estimate the concentration errors. The maximum relative deviation from the nominal concentration is found to be ≈ 20% in all experiments performed. Fig D, Overview of the image analysis procedure. (A) A composite brightfield and fluorescence image, cropped to display only the microchamber contents. The program workflow is demonstrated by focusing on the red highlighted area of the picture, a region with dimensions 21.6×12.4 *μ*m^2^. (B) Once the brightfield image is imported, contrast is enhanced and resolution increased. (C) Background detection is performed via a hybrid method combining adaptive thresholding and geometry information. (D) Cell markers are created using gradient information and geometric priors, refined using the watershed method. (E) A statistical classifier is used to remove mis-segmented cells. (F-I) Using the maximum overlap method, cell lineages are reconstructed (see also Fig D in S1 File). A cell division event is highlighted in (F-G); and propagated forward in (H-I). The user can correct tracking errors manually in the application. Fig E, Example of a cell lineage extracted using the segmentation software. The lineage is first automatically calculated by using the maximum overlap method on the segmented cells, as described in the main text. The segmentation method is conservative in detecting cell divisions, which means that already divided cells may be detected as a single cell for a few frames longer. This explains the observed cell division timings in the above lineage tree. After this step a correction heuristic is applied which finds potential mother-daughter mismatches by searching for fluorescence fluctuations twice as large as the calculated noise in a typical trajectory. For presentational clarity any branches which do not reach the final frame (due to mismatches) were manually edited out of the above plot. Fig F, Bulk analysis of gene induction by AHL using plate reader measurements. (A) Background subtracted absorbance of growing bacterial cultures for AHL concentrations ranging from 0 nM to 100 nM. (B) Corresponding background subtracted fluorescence intensities for the different AHL concentrations. (C) Maximum gene expression rate *α*_*max*_ obtained for the different AHL concentrations as explained in the main text. The solid line is a fit with a Hill curve with Hill exponent *n* = 0.97±0.08 and induction threshold *K* = 13.9±1.7 nM.
(PDF)

## References

[1] Fuqua C, Greenberg EP. Listening in on bacteria: acyl-homoserine lactone signalling. Nat Rev Mol Cell Bio. 2002;3(9):685–95. 12209128 10.1038/nrm907

[2] Waters CM, Bassler BL. Quorum Sensing: Cell-to-Cell Communication in Bacteria. Annu Rev Cell Dev Bi. 2005;21(1):319–346. 10.1146/annurev.cellbio.21.012704.131001 16212498

[3] Weiss R, Knight TF Jr. Engineered Communications for Microbial Robotics In: Condon AE, Rozenberg G, editors. DNA Computing, 6th International Workshop on DNA-Based Computers, DNA6. vol. 2054 of Lecture Notes in Computer Science. Springer; 2000 p. 1–16.

[4] You LC, Cox RS, Weiss R, Arnold FH. Programmed population control by cell-cell communication and regulated killing. Nature. 2004;428(6985):868–871. 15064770 10.1038/nature02491

[5] Balagadde FK, You LC, Hansen CL, Arnold FH, Quake SR. Long-term monitoring of bacteria undergoing programmed population control in a microchemostat. Science. 2005;309(5731):137–140. 10.1126/science.1109173 15994559

[6] Basu S, Gerchman Y, Collins CH, Arnold FH, Weiss R. A synthetic multicellular system for programmed pattern formation. Nature. 2005;434(7037):1130–4. 15858574 10.1038/nature03461

[7] Sohka T, Heins R, Phelan R, Greisler J, Townsend C, Ostermeier M. An externally tunable bacterial band-pass filter. P Natl Acad Sci USA. 2009;106(25):10135–10140. 10.1073/pnas.0901246106 PMC2700907 19502423

[8] Danino T, Mondragón-Palomino O, Tsimring L, Hasty J. A synchronized quorum of genetic clocks. Nature. 2010;463(7279):326–330. 20090747 10.1038/nature08753 PMC2838179

[9] Tabor JJ, Salis HM, Simpson ZB, Chevalier AA, Levskaya A, Marcotte EM, et al A Synthetic Genetic Edge Detection Program. Cell. 2009 6;137(7):1272–1281. 19563759 10.1016/j.cell.2009.04.048 PMC2775486

[10] Tamsir A, Tabor JJ, Voigt CA. Robust multicellular computing using genetically encoded NOR gates and chemical ‘wires’. Nature. 2011;469(7329):212–215. 21150903 10.1038/nature09565 PMC3904220

[11] Weitz M, Mückl A, Kapsner K, Berg R, Meyer A, Simmel FC. Communication and Computation by Bacteria Compartmentalized within Microemulsion Droplets. J Am Chem Soc. 2014;136(1):72–75. 10.1021/ja411132w 24358940

[12] Elowitz MB, Levine AJ, Siggia ED, Swain PS. Stochastic gene expression in a single cell. Science. 2002;297(5584):1183–1186. 10.1126/science.1070919 12183631

[13] Golding I, Paulsson J, Zawilski S, Cox E. Real-time kinetics of gene activity in individual bacteria. Cell. 2005;123(6):1025–1036. 16360033 10.1016/j.cell.2005.09.031

[14] Kaern M, Elston TC, Blake WJ, Collins JJ. Stochasticity in gene expression: from theories to phenotypes. Nat Rev Genet. 2005;6(6):451–464. 15883588 10.1038/nrg1615

[15] Raj A, van Oudenaarden A. Nature, Nurture, or Chance: Stochastic Gene Expression and Its Consequences. Cell. 2008;135(2):216–226. 18957198 10.1016/j.cell.2008.09.050 PMC3118044

[16] Munsky B, Neuert G, van Oudenaarden A. Using gene expression noise to understand gene regulation. Science. 2012;336(6078):183–7. 10.1126/science.1216379 22499939 PMC3358231

[17] Tsimring LS. Noise in biology. Rep Prog Phys. 2014;77(2):026601 Available from: http://iopscience.iop.org/0034-4885/77/2/026601. 24444693 10.1088/0034-4885/77/2/026601 PMC4033672

[18] Silander OK, Nikolic N, Zaslaver A, Bren A, Kikoin I, Alon U, et al A Genome-Wide Analysis of Promoter-Mediated Phenotypic Noise in Escherichia coli. PLoS Genet. 2012;8(1):e1002443 Available from: 10.1371/journal.pgen.1002443. 10.1371/journal.pgen.1002443 22275871 PMC3261926

[19] Paulsson J. Models of stochastic gene expression. Phys Life Rev. 2005;2(2):157–175. 10.1016/j.plrev.2005.03.003

[20] Friedman N, Cai L, Xie XS. Linking stochastic dynamics to population distribution: An analytical framework of gene expression. Phys Rev Lett. 2006;97(16). 10.1103/PhysRevLett.97.168302 17155441

[21] Huh D, Paulsson J. Non-genetic heterogeneity from stochastic partitioning at cell division. Nat Genet. 2011;43(2):95–100. 21186354 10.1038/ng.729 PMC3208402

[22] Huh D, Paulsson J. Random partitioning of molecules at cell division. P Natl Acad Sci USA. 2011;108(36):15004–15009. 10.1073/pnas.1013171108 PMC3169110 21873252

[23] Cox CD, Peterson GD, Allen MS, Lancaster JM, McCollum JM, Austin D, et al Analysis of noise in quorum sensing. Omics: a journal of integrative biology. 2003;7(3):317–34. 10.1089/153623103322452422 14583119

[24] Tanouchi Y, Tu D, Kim J, You L. Noise Reduction by Diffusional Dissipation in a Minimal Quorum Sensing Motif. PLoS Computational Biology. 2008;4(8):e1000167 10.1371/journal.pcbi.1000167 18769706 PMC2507755

[25] Long T, Tu KC, Wang Y, Mehta P, Ong NP, Bassler BL, et al Quantifying the Integration of Quorum-Sensing Signals with Single-Cell Resolution. PLoS Biol. 2009;7(3):e68 10.1371/journal.pbio.1000068 19320539 PMC2661960

[26] Wang Y, Tu KC, Ong NP, Bassler BL, Wingreen NS. Protein-level fluctuation correlation at the microcolony level and its application to the Vibrio harveyi quorum-sensing circuit. Biophysical journal. 2011;100(12):3045–53. 21689539 10.1016/j.bpj.2011.05.006 PMC3123921

[27] Locke JCW, Elowitz MB. Using movies to analyse gene circuit dynamics in single cells. Nat Rev Microbiol. 2009;7(5):383–392. 19369953 10.1038/nrmicro2056 PMC2853934

[28] Groisman A, Lobo C, Cho H, Campbell JK, Dufour YS, Stevens AM, et al A microfluidic chemostat for experiments with bacterial and yeast cells. Nat Methods. 2005;2(9):685–689. 16118639 10.1038/nmeth784

[29] Bennett MR, Hasty J. Microfluidic devices for measuring gene network dynamics in single cells. Nat Rev Genet. 2009;10(9):628–638. 19668248 10.1038/nrg2625 PMC2931582

[30] Yan L, Allen MS, Simpson ML, Sayler GS, Cox CD. Direct quantification of N-(3-oxo-hexanoyl)-L-homoserine lactone in culture supernatant using a whole-cell bioreporter. Journal of microbiological methods. 2007;68(1):40–5. 16916554 10.1016/j.mimet.2006.06.002

[31] Hense BA, Kuttler C, Müller J, Rothballer M, Hartmann A, Kreft JU. Does efficiency sensing unify diffusion and quorum sensing?. Nat Rev Microbiol. 2007;5(3):230–9. 17304251 10.1038/nrmicro1600

[32] West SA, Winzer K, Gardner A, Diggle SP. Quorum sensing and the confusion about diffusion. Trends Microbiol. 2012;20(12):586–594. 10.1016/j.tim.2012.09.004 23084573

[33] Cormack B, Valdivia R, Falkow S. FACS-optimized mutants of the green fluorescent protein (GFP). Gene. 1996;173(1):33–38. 8707053 10.1016/0378-1119(95)00685-0

[34] Dertinger SKW, Chiu DT, Jeon NL, Whitesides GM. Generation of Gradients Having Complex Shapes Using Microfluidic Networks. Anal Chem. 2001;73(6):1240–1246. 10.1021/ac001132d

[35] Wang Q, Niemi J, Tan CM, You L, West M. Image segmentation and dynamic lineage analysis in single-cell fluorescence microscopy. Cytom Part A. 2010;77A(1):101–110. Available from: http://doi.wiley.com/10.1002/cyto.a.20812. 10.1002/cyto.a.20812 PMC2797831 19845017

[36] Rämö P, Sacher R, Snijder B, Begemann B, Pelkmans L. CellClassifier: supervised learning of cellular phenotypes. Bioinformatics. 2009 11;25(22):3028–3030. 10.1093/bioinformatics/btp524 19729371

[37] Urbanowski ML, Lostroh CP, Greenberg EP. Reversible acyl-homoserine lactone binding to purified Vibrio fischeri LuxR protein. J Bacteriol. 2004;186(3):631–7. 10.1128/JB.186.3.631-637.2004 14729687 PMC321501

[38] Haseltine EL, Arnold FH. Implications of Rewiring Bacterial Quorum Sensing. Applied and Environmental Microbiology. 2008;74(2):437–445. 10.1128/AEM.01688-07 18039819 PMC2223271

[39] Carbonell-Ballestero M, Duran-Nebreda S, Montanez R, Sole R, Macia J, Rodriguez-Caso C. A bottom-up characterization of transfer functions for synthetic biology designs: lessons from enzymology. Nucleic Acids Res. 2014;42(22):14060–14069. 10.1093/nar/gku964 25404136 PMC4267673

[40] Koch AL. The logarithm in biology 1. Mechanisms generating the log-normal distribution exactly. J Theor Biol. 1966 11;12(2):276–290. 10.1016/0022-5193(66)90119-6 5972197

[41] Koch AL. The logarithm in biology: II. Distributions simulating the log-normal. J Theor Biol. 1969 5;23(2):251–268. 10.1016/0022-5193(69)90040-X 5821531

[42] Berg OG. A model for the statistical fluctuations of protein numbers in a microbial population. J Theor Biol. 1978;71(4):587–603. 10.1016/0022-5193(78)90326-0 96307

[43] Ozbudak EM, Thattai M, Kurtser I, Grossman AD, van Oudenaarden A. Regulation of noise in the expression of a single gene. Nat Genet. 2002 5;31(1):69–73. 10.1038/ng869 11967532

[44] Shahrezaei V, Swain PS. Analytical distributions for stochastic gene expression. Proc Natl Acad Sci USA. 2008 11;105(45):17256–17261. 10.1073/pnas.0803850105 18988743 PMC2582303

[45] Taniguchi Y, Choi PJ, Li GW, Chen H, Babu M, Hearn J, et al Quantifying E. coli proteome and transcriptome with single-molecule sensitivity in single cells. Science. 2010 7;329(5991):533–538. 10.1126/science.1188308 20671182 PMC2922915

[46] Hense BA, Müller J, Kuttler C, Hartmann A. Spatial heterogeneity of autoinducer regulation systems. Sensors. 2012;12(4):4156–4171. PMCID: PMC3355405. 10.3390/s120404156 22666024 PMC3355405

[47] Dilanji GE, Langebrake JB, De Leenheer P, Hagen SJ. Quorum activation at a distance: spatiotemporal patterns of gene regulation from diffusion of an autoinducer signal. J Am Chem Soc. 2012 3;134(12):5618–5626. 10.1021/ja211593q 22372494

[48] Choi WS, Ha D, Park S, Kim T. Synthetic multicellular cell-to-cell communication in inkjet printed bacterial cell systems. Biomaterials. 2011 4;32(10):2500–2507. 10.1016/j.biomaterials.2010.12.014 21208654

