# Supplementary material for: Single Cell Analysis of a Bacterial Sender-Receiver System
Source: PLoS One. 2016 Jan 25;11(1):e0145829. doi: 10.1371/journal.pone.0145829 (PMC4726700; doi:10.1371/journal.pone.0145829)
Supplement: S1 File — Text A, Image processing. Text B, Gene expression noise. Text C, Sender—receiver system. Fig A, Schematic overview of the bacterial sender-receiver system. Sender cells: As indicated, in the presence of IPTG repressor protein LacI is not bound to the lac promoters PLacUV5 on the bacterial genome and PT7lac on the sender plasmid. T7 RNA polymerase is then expressed, which in turn leads to the expression of AHL synthase LuxI and fluorescent reporter protein RFP from the plasmid. LuxI catalyzes the production of the quorum sensing signal N-3-oxo-C6-homoserine lactone (AHL), which can freely pass through the bacterial cell wall. Receiver cells constitutively express activator LuxR from the receiver plasmid. In the presence of AHL, LuxR activates GFP expression, which is under the control of the lux promoter Plux. In the first set of experiments in the main paper, only receiver cells are used and AHL is manually added to the culture medium to induce gene expression. Fig B, Microfluidic chemostats. (A) The microfluidic chemostat consists of a gradient mixer (adopted from Ref. 55 of the main paper), which generates linear concentration gradients of chemicals supplied through inlets 1 and 2, respectively. Eight gradient exits are connected to a total of 2 × 8 microfluidic channels, which contain trapping regions for bacteria (similar to Ref. 8 of the main paper). In the experiments, the concentration of AHL was varied in 1 nM steps in the range 0–21 nM, and in 10 nM steps in the range 20–90 nM. (B) Top view of a supply channel (blue) with trap region (grey). (C) Side view (not drawn to scale) showing the reduced height of the trap region, which only allows bacterial growth in a single layer. Fig C, Calibration of the gradient mixer system. We performed a series of calibraton experiments (with flow rates 40, 80, 160 and 320 μl/h) to evaluate the quality of the concentration gradient generated by the microfluidic mixer shown in Fig B in S1 File. In these experiments the right [file pone.0145829.s001.pdf]

Ramalho et al., Single cell analysis of a bacterial sender-receiver system

S1 File

- Supplementary Texts:

A Image processing
B Gene expression noise
C Sender - receiver system

- Supplementary Figures A - F

A Image processing

Background

We developed a custom software application to automate the single cell analysis of brightfield images. Our goal was to strike a balance between accuracy, performance and ease of use. To do so, we evaluated several existing applications (CellProfiler [1], CellTracer [2], http://cellcognition.org/etc) and implemented the most useful features. Source code and binaries freely available here. We prioritized simplicity over flexibility by using a fixed image processing pipeline. We expect labs with similar workflows will be able to use our application with a minimal learning curve. In the following sections, a brief outline of the image analysis algorithm is given along with a rationale for the choice of the used methods.

Image preprocessing

The first step is image preprocessing. The user may define a subsection of the image stack to analyze which is then enhanced by applying well known filters: contrast enhancement, noise reduction, sharpening and resolution increase. Contrast is enhanced by calculating the brightness histogram; discarding all brightness values beneath a user-defined threshold and rescaling the brightness values such that the histogram now spans the full brightness range. Noise reduction is accomplished by the nonlocal means filter implemented in the OpenCV library [3]. The image is sharpened by subtracting the lowpass filtered image. Finally, the accuracy of many subsequent pipeline steps is increased if we work with a higher resolution image. Thus, the user has the option to double or quadruple the image resolution at this step.

Background detection

We found that optimal performance was achieved by combining the output of several simple thresholding methods. First, a global brightness threshold may be set by the user. Second, a local brightness threshold is used, where a pixel is marked as background if its brightness is above the local brightness average by a pre specified amount. The local average is computed around a window of pre-specified size which should match the typical length scale of the smallest axis of a cell. Finally, large areas with no cells which might elude the previous two methods are detached by employing a method similar to Wang et al [2], where prominent edges are detected and enlarged to outline the probable location of the cells; the complement of that area is marked as background.

Cell detection

Given specified maximum bounds on width and height, cell markers are created by a multi-step procedure. Initially all connected regions of non-background pixels are assigned to a unique marker. Each region too big to be a single cell is then further segmented based on the brightness profile and cell geometry. This process uses a likelihood function, which assigns a likelihood for each of pixel to be part of the inside of a cell, taking into account the brightness, the brightness gradient, and the cell geometry (by using the distance transform). New markers are then assigned to connected regions of pixels above a certain likelihood threshold. This threshold is chosen to be the minimum value such that the regions of all markers obey the specified bound. The resulting regions are then expanded and refined using the watershed algorithm.

Cell classification

Often, objects which are not cells are present in the microscopy image but are still detected as such due to similar brightness profiles as real cells. To overcome this problem, an optional final step of the operation pipeline entails the training of a support vector machine (SVM) to distinguish interesting cells from such outliers. This method has previously been used to distinguish cell phenotypes with success [4].

The process begins by taking each connected area of segmented pixels and calculating features summarizing its geometry and brightness, which will become a high dimensional data point d_i for the training set. Each point is selected by the user, who also marks each label as correct or incorrect directly on the user interface thereby associating each datapoint with a class $\mathcal{C} \in 0, 1$. The SVM algorithm then applies a nonlinear transformation to the feature space such that a hyperplane separating the two classes of points can be found. The implementation found in libSVM [5] has been used here. Empirically we determined that a dataset with around 50 points provides good classification performance. By visual inspection the user may validate the results and edit the training data to avoid over or under fitting.

Lineage tracking

In order to track cells in time, a frame by frame tracking procedure was adopted. In each frame we determine a cell's parent by calculating the overlap between its assigned pixels and the pixels of detected cells in the previous frame. The cell label from the previous frame which maximizes this overlap is then set as the parent. If l_i^t is a boolean vector where pixels are marked as 1 if they belong to the ith label at frame t and 0 otherwise; the parent label for that label is defined as

$$p_{i,t} = \underset{j \in \text{labels}(t-1)}{\arg\max} \sum_{\text{pixels}} l_j^{t-1} l_i^t \ .$$

This method was compared to the minimization of the distance between the center of mass of a cell and those of its ancestors. If c_i^t is a 2d vector containing the center of mass of the ith label at frame t, then

$$p_{i,t} = \underset{j \in \text{labels}(t-1)}{\arg\min} \ c_j^{t-1} . c_i^t \ .$$

Empirically the maximum overlap method performed better than the center of mass distance for all datasets we tried. As before, the user can inspect the generated trajectories and manually correct the lineage in case of error via a graphical user interface. This allowed us to automatically extract lineage data and single cell fluorescence trajectories as a function of time.

Single cell trajectories

Fluorescence is extracted by summing measured intensity at each pixel for each channel. In this case we define

$$p^k = \sum_{i,j \in \mathcal{L}(k)} f_{i,j}$$

with $f_{i,j}$ the intensity of a given pixel in the kth detected label, denoted by $\mathcal{L}(k)$. The area A of a cell can also be calculated by summing over all pixels in a label.

Once we have obtained the time trajectories of $p^k(t)$ and $A^k(t)$, we can fit them using a smoothing spline to be able to calculate derivatives and then use $\alpha^k(t) \sim \dot{p}^k(t)/A^k(t)$, as in the previous sections.

Image analysis for sender-receiver experiments

The image analysis methodology for these experiments is largely parallel to the one for the receiver-only experiments. The cells are segmented using the image analysis program, and RFP and GFP fluorescences are extracted. A bivariate Gaussian mixture model is fit to the final frame to discover senders and receivers, and their lineage is backtracked throughout the experiment to obtain their induction trajectories. From these trajectories, GFP and RFP expression rates can be extracted.

Comparison with other methods

Our single cell analysis yielded significantly better results than a naive analysis based on the public domain image processing program ImageJ [6]. Such an analysis was based on a thresholding procedure where all pixels of a brightfield image with brightness below a certain threshold are assigned as 'cell pixels' and that integrated area is taken as a proxy for cell mass. This procedure incurs the systematic error of adding the mass of dark objects to the cell mass, which means that the determined area values are not fully compatible with the determined fluorescence (objects which are not cells do not fluoresce). The response function obtained with this procedure appeared less accurate (Figure S3), however it was nevertheless qualitatively similar to the response shown in Fig. 1C of the main paper with the more elaborate procedure described above.

B Gene expression noise

Noise quantification

We analyzed the noise in our data on different levels. At the lowest level there are fluctuations in intensity level between the individual pixels corresponding to a cell. These fluctuations may be partially due to experimental error, but also inherent to inhomogeneity in gene expression in the cell. We calculate the average and standard deviation of a single cell's fluorescence as

$$\langle p^k \rangle = \sum_{i,j \in \mathcal{L}(k)} p_{i,j}/a^k \tag{1}$$

(using the notation introduced above) and

$$\sigma^2_{p^k} = \sum_{i,j \in \mathcal{L}(k)} (p_{i,j} - \langle p^k \rangle)^2/a^k \ , \tag{2}$$

where $a^k = \sum_{i,j \in \mathcal{L}(k)}$. Then there is the noise arising from heterogeneity between different individuals in a population, which can be calculated from the previous values,

$$\bar{p} = \sum_k \langle p^k \rangle / N \ , \tag{3}$$

$$\sigma^2_p = \sum_k (\langle p^k \rangle - \bar{p})^2/N \ . \tag{4}$$

Finally there is the temporal evolution noise. For each temporal trajectory, we can calculate a mean and standard deviation via the use of a smoothing filter. This relates the temporal noise for a single cell.

C Sender-receiver system

The evolution equations

$$\frac{d}{dt}[\text{LuxI}](t) = \alpha_l\, rN(t) - \lambda\,[\text{LuxI}](t). \tag{5}$$

and

$$\frac{d}{dt}[\text{AHL}](t) = \alpha_a[\text{LuxI}](t) - C\,[\text{AHL}](t) \tag{6}$$

given in the main text can be solved analytically for exponential cell growth $N(t) = N_o e^{\gamma t}$. The solution to the first equation is given by:

$$[\text{LuxI}](t) = \frac{N_0\alpha_l r}{\gamma+\lambda}\left(e^{\gamma t} - e^{-\lambda t}\right) \tag{7}$$

Insertion into Eq.(7) can then be solved to yield:

$$[\text{AHL}](t) = \frac{N_0\alpha_l r}{(\gamma+\lambda)(C+\gamma)(C-\lambda)}\left[C\left(e^{\gamma t} - e^{-\lambda t}\right) + \gamma\left(e^{-Ct} - e^{-\lambda t}\right) + \lambda\left(e^{-Ct} - e^{\gamma t}\right)\right] \tag{8}$$

Expanding the exponentials for low values of C, γ, λ (up to terms of order t^2) results in:

$$[\text{AHL}](t) \approx \frac{1}{2}N_0\alpha_l\alpha_a r t^2, \tag{9}$$

as given in the main text.

References

1. Carpenter A, Jones T, Lamprecht M, Clarke C, Kang I, Friman O, et al. CellProfiler: image analysis software for identifying and quantifying cell phenotypes. Genome Biol. 2006;7(10):R100.

2. Wang Q, Niemi J, Tan CM, You L, West M. Image segmentation and dynamic lineage analysis in single-cell fluorescence microscopy. Cytom Part A. 2010;77A(1):101–110. Available from: `http://doi.wiley.com/10.1002/cyto.a.20812`.

3. Bradski G. The OpenCV Library. Dr Dobb's Journal of Software Tools. 2000.

4. Rämö P, Sacher R, Snijder B, Begemann B, Pelkmans L. CellClassifier: supervised learning of cellular phenotypes. Bioinformatics. 2009 Nov;25(22):3028–3030.

5. Chang CC, Lin CJ. LIBSVM: A library for support vector machines. ACM Trans Intell Syst Technol. 2011;2:27:1–27:27.

6. Schneider CA, Rasband WS, Eliceiri KW. NIH Image to ImageJ: 25 years of image analysis. Nat Meth. 2012;9(7):671–675.

Supplementary Figures

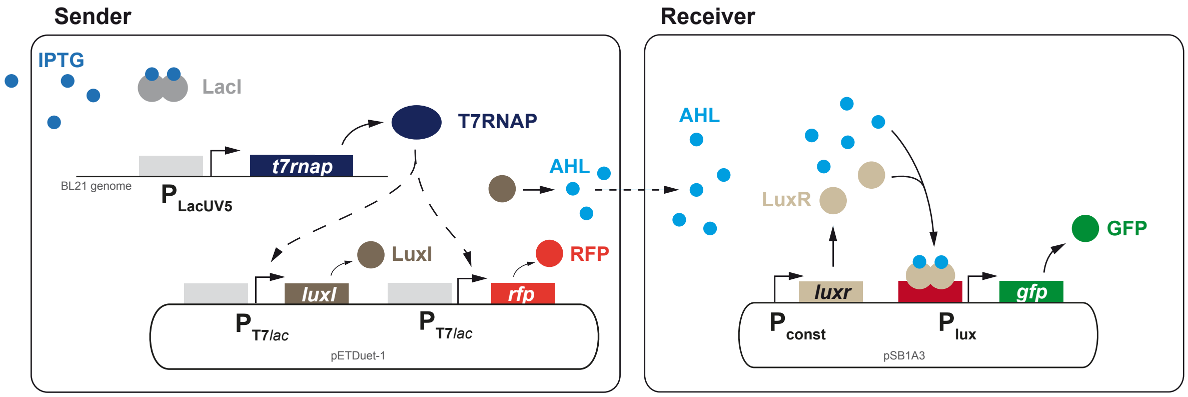

Fig A. Schematic overview of the bacterial sender-receiver system. *Sender cells*: As indicated, in the presence of IPTG repressor protein LacI is not bound to the lac promoters P_{LacUV5}on the bacterial genome and P_{T7lac} on the sender plasmid. T7 RNA polymerase is then expressed, which in turn leads to the expression of AHL synthase LuxI and fluorescent reporter protein RFP from the plasmid. LuxI catalyzes the production of the quorum sensing signal N-3-oxo-C6-homoserine lactone (AHL), which can freely pass through the bacterial cell wall. *Receiver cells* constitutively express activator LuxR from the receiver plasmid. In the presence of AHL, LuxR activates GFP expression, which is under the control of the lux promoter P_{lux}. In the first set of experiments in the main paper, only receiver cells are used and AHL is manually added to the culture medium to induce gene expression.

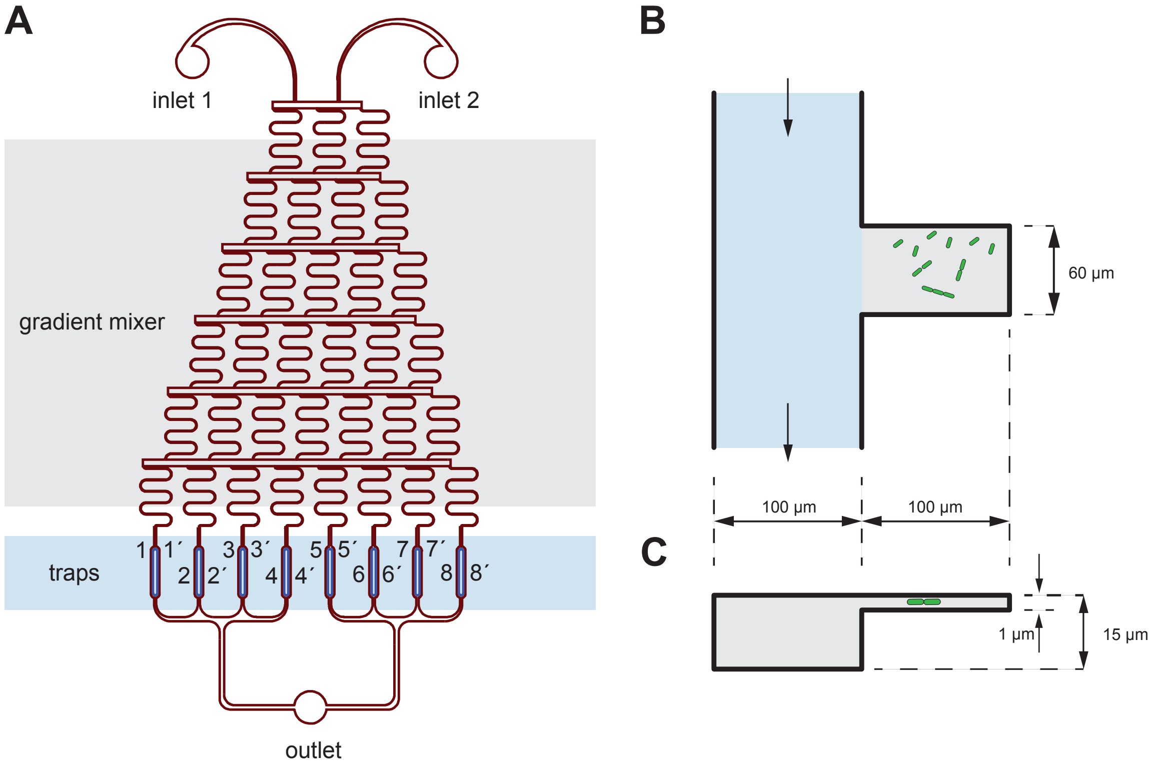

Fig B. Microfluidic chemostats. (A) The microfluidic chemostat consists of a gradient mixer (adopted from Ref. 55 of the main paper), which generates linear concentration gradients of chemicals supplied through inlets 1 and 2, respectively. Eight gradient exits are connected to a total of 2 x 8 microfluidic channels, which contain trapping regions for bacteria (similar to Ref. 8 of the main paper). In the experiments, the concentration of AHL was varied in 1 nM steps in the range 0 – 21 nM, and in 10 nM steps in the range 20 – 90 nM. (B) Top view of a supply channel (blue) with trap region (grey). (C) Side view (not drawn to scale) showing the reduced height of the trap region, which only allows bacterial growth in a single layer.

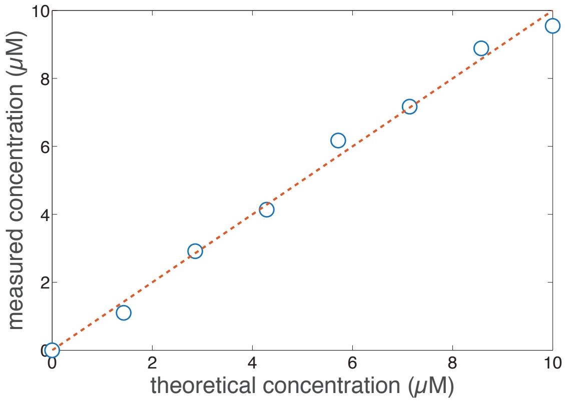

Fig C. Calibration of the gradient mixer system. We performed a series of calibraton experiments (with flow rates 40, 80, 160 and 320 μl/h) to evaluate the quality of the concentration gradient generated by the microfluidic mixer shown in Fig. S2. In these experiments the right reservoir was loaded with buffer solution containing 10 μM fluorescein and the left reservoir with pure buffer (0 μM). After establishment of a steady gradient, we measured the fluorescence in the trap regions. The background-subtracted fluorescence values were then plotted against the nominal concentrations expected for the traps. As shown in the figure (which is obtained for the 160μl/h case), indeed a linear concentration gradient is generated. A linear regression fit to these values (fixed at 0μM) allows us to estimate the concentration errors. The maximum relative deviation from the nominal concentration is found to be $\approx 20\%$ in all experiments performed.

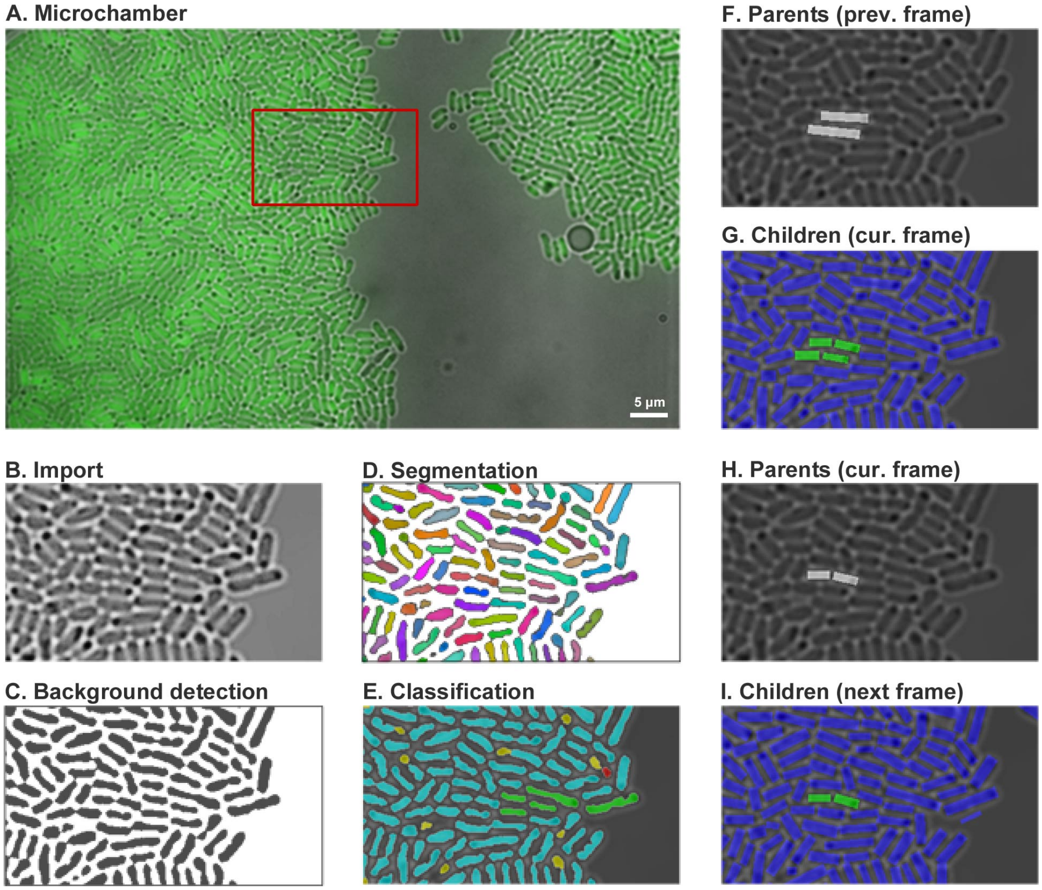

Fig D. Overview of the image analysis procedure. (A) A composite brightfield and fluorescence image, cropped to display only the microchamber contents. The program workflow is demonstrated by focusing on the red highlighted area of the picture, a region with dimensions $21.6 \times 12.4\ \mu m^2$. (B) Once the brightfield image is imported, contrast is enhanced and resolution increased. (C) Background detection is performed via a hybrid method combining adaptive thresholding and geometry information. (D) Cell markers are created using gradient information and geometric priors, refined using the watershed method. (E) A statistical classifier is used to remove mis-segmented cells. (F-I) Using the maximum overlap method, cell lineages are reconstructed (see also Fig. S4). A cell division event is highlighted in (F-G); and propagated forward in (H-I). The user can correct tracking errors manually in the application.

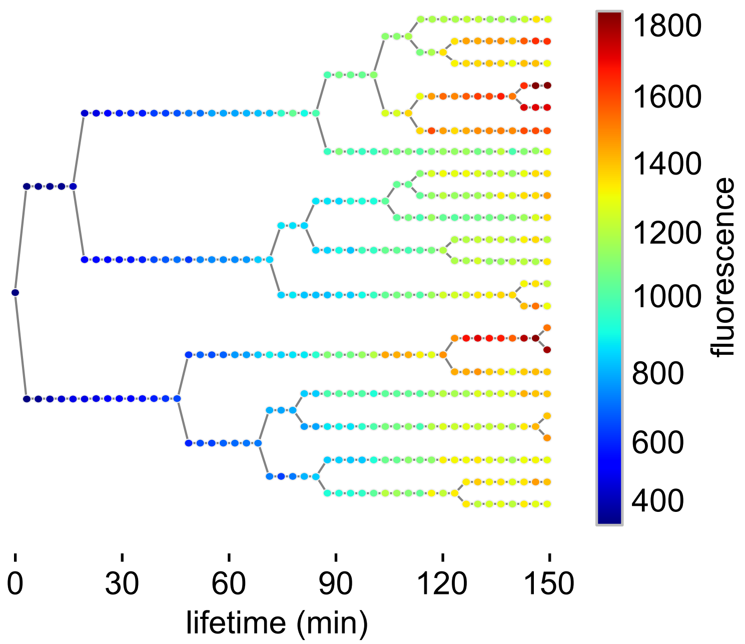

Fig E. Example of a cell lineage extracted using the segmentation software. The lineage is first automatically calculated by using the maximum overlap method on the segmented cells, as described in the main text. The segmentation method is conservative in detecting cell divisions, which means that already divided cells may be detected as a single cell for a few frames longer. This explains the observed cell division timings in the above lineage tree. After this step a correction heuristic is applied which finds potential mother-daughter mismatches by searching for fluorescence fluctuations twice as large as the calculated noise in a typical trajectory. For presentational clarity any branches which do not reach the final frame (due to mismatches) were manually edited out of the above plot.

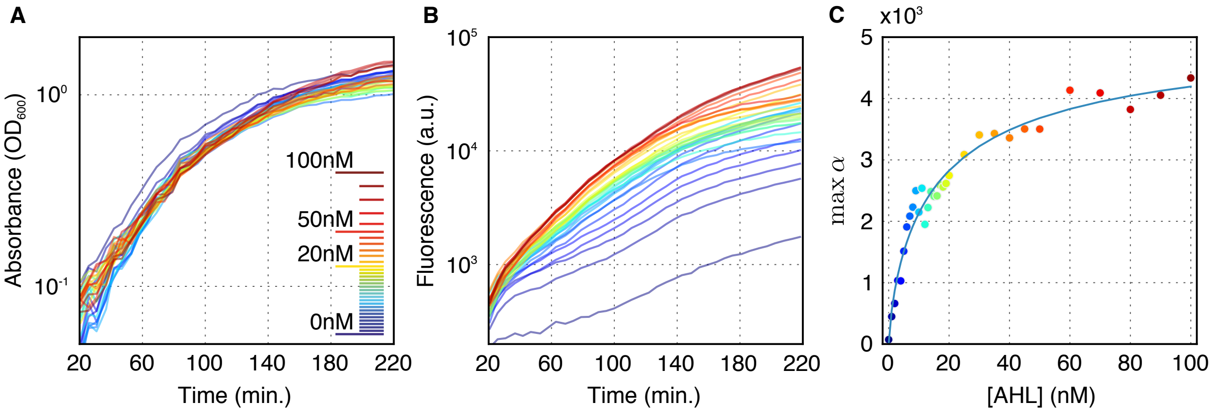

Fig F. Bulk analysis of gene induction by AHL using plate reader measurements. (A) Background subtracted absorbance of growing bacterial cultures for AHL concentrations ranging from 0 nM to 100 nM. (B) Corresponding background subtracted fluorescence intensities for the different AHL concentrations. (C) Maximum gene expression rate α_{max} obtained for the different AHL concentrations as explained in the main text. The solid line is a fit with a Hill curve with Hill exponent $n = 0.97 \pm 0.08$ and induction threshold $K = 13.9 \pm 1.7$ nM.
